# Improving the Adhesive Properties by Sandblasting the Surface with Copper Slag and Glass Beads

**DOI:** 10.3390/ma18081746

**Published:** 2025-04-10

**Authors:** Jacek Ogrodniczek, Anna Rudawska, Agnieszka Skoczylas, Sławomir Kocira

**Affiliations:** 1 Faculty of Production Engineering, University of Life Sciences in Lublin, Głęboka 28, 20-612 Lublin, Poland; jacek.ogrodniczek@up.lublin.pl; 2 Faculty of Mechanical Engineering, Lublin University of Technology, Nadbystrzycka 36, 20-618 Lublin, Poland; a.rudawska@pollub.pl (A.R.); a.skoczylas@pollub.pl (A.S.); 3 Faculty of Agriculture and Technology, University of South Bohemia in České Budějovice, 370 05 České Budějovice, Czech Republic

**Keywords:** glass, aluminium alloy, adhesive joints, sandblasting

## Abstract

The aim of the research was to examine the effect of sandblasting the adherent surfaces on the shear strength of aluminium alloy–glass adhesive joints. An EN AW-1050A aluminium alloy and non-hardened soda-lime glass were used as samples. The sandblasting materials in the study were copper slag and glass beads. Due to the brittleness of the glass, an adhesive joint design was created to allow strength testing. The adhesive joints took the form of lap joints, where a glass sample was placed between two aluminium samples. The length of the glass sample corresponded to the length of the adhesive joint overlap. The bonding process of the glass samples was carried out in two stages in order to maintain the dimensions of the joint. The adhesive used in the test was a two-component epoxy adhesive prepared on the basis of bisphenol A and a polyamide curing agent mixed at a ratio of 80 g of curing agent per 100 g of epoxy resin. For each sandblasting agent, adhesive joints consisting of three variations of surface treatments were made: sandblasted aluminium alloy-sandblasted glass, sandblasted aluminium alloy-untreated glass, untreated aluminium alloy-sandblasted glass. The distance between the nozzle and treated surface was 10 ± 1 cm, and the pressure of sandblasting was 0.6 MPa. One sample of each material differing in surface preparation was selected for a topography analysis. The shear strength tests performed showed that sandblasting increased the strength of the adhesive joints of the glass and aluminium alloy. The highest strength was obtained for joints where both materials were prepared using copper slag sandblasting. This was confirmed through statistical analysis. Only for this type of joint, statistical significance relative to the other adhesive bonding variants was obtained.

## 1. Introduction

The surface preparation of materials has a significant impact on the strength of adhesive joints. There are several operations to change the surface properties, including mechanical, chemical and electrochemical methods [1,2,3]. When creating adhesive joints, the most commonly used method is the mechanical method, which creates a surface with a rough structure. The choice of this method is due to the existence of the mechanical theory of adhesion, which says that the adhesive particles penetrate the unevenness on the surface of the material and then anchor to form an adhesive joint that is not destructed under the influence of external loads [4,5,6,7]. Excessive roughness of the surface of the material may result in a decrease in the strength of the adhesive bond. The critical value of roughness depends on many parameters such as the adhesive type, adhesive density, adhesive layer geometry and stress type. The surface roughness affects the way the adhesive is distributed. Excessive roughness may contribute to an uneven distribution of the adhesive over the surface, creating voids [8,9,10].

Sandblasting is classified as abrasive blasting. This treatment consists of ejecting the abrasive agent at high speed onto the work surface under the influence of compressed air [3]. Many sandblasting agents are available, varying in applications and the size of a single grain. Sandblasting agents may include electrocorundum, copper slag, nut shells, glass beads or soda ash. The sandblasting operation allows one to clean the surface from impurities, to give aesthetic qualities or deliberately change the geometry of the treated surface [11,12,13]. After the sandblasting operation, the surface quality is similar to that obtained after the grinding treatment. The advantage of this method is that a rough and homogeneous structure is obtained on the surface of the material. Sandblasting is often used prior to the application of coatings to the surface of the material; therefore, it is possible to use this method in adhesive bonding technology. Some abrasives, when subjected to high-velocity discharge from the nozzle, can combine with the material to form surface contaminants. This phenomenon must be taken into account to avoid technological errors in adhesive joints. However, for most cases of surface preparation using sandblasting, it is sufficient to wipe the surface with a cleaning agent [14,15,16,17].

Glass, due to its brittle structure, is a material that is difficult to work with. In order to change the structure of the glass surface, it is possible to employ chemical methods that allow the surface of the material to be etched, to apply coatings or to use thermal treatment or abrasive blasting [18,19].

The last type of glass surface treatment is most commonly used for aesthetic purposes. However, the altered structure of the glass surface as a result of sandblasting treatment may contribute to an increase in the adhesive forces [9]. Due to the lack of research using the sandblasting of glass, the article opts for this type of surface preparation of this material. For the research, glass beads and copper slag were chosen because of their hardness on the Mohs scale, which allows the surface structure to be altered without causing major damage to the glass material.

Rudawska et al. [20] conducted an experiment in which the effect of sandblasting pressure on the surface of an aluminium alloy on the strength of single-lap adhesive joints was investigated. In this study, three types of aluminium alloy and one sandblasting agent, EB F54 aloxite, were selected. The surfaces of the samples were sandblasted at pressures of 0.41 MPa, 0.51 MPa and 0.56 MPa. The adhesive bond was a compound of a bisphenol A-based epoxy resin and a polyamide hardener, mixed in a ratio of 100:80. This paper shows that the aluminium alloy surface treatment with sandblasting affects the change in the adhesive joint strength.

Machalická and Eliášová [21] presented a paper where they investigated the influence of various factors on the mechanical properties of adhesive joints in glass structures. Glass–steel, glass–stainless steel and glass–aluminium alloy joints were created. The adhesives used in the work were a single-component polyurethane adhesive, a two-component polyurethane adhesive, a two-component acrylic adhesive and a UV-curable adhesive. The surface of the glass samples in the glass–steel joints was prepared using sandblasting. To carry out the tests, the authors constructed a specimen grip to conduct the strength tests. Two rectangular glass samples were bonded to the metal sample. The tests showed an increase in strength after the glass samples were treated with sandblasting.

The aim of the paper was to prepare adhesive joints formed from materials prepared in different variants using sandblasting treatment, to compare the strength of the formed adhesive joints and to demonstrate the effect of sandblasting treatment on the strength of glass—aluminium alloy adhesive joints.

## 2. Materials and Methods

### 2.1. Material Characteristics

The first material used for the test was an aluminium alloy with the designation EN AW-1050A. It is referred to as a pure aluminium alloy due to its very high aluminium content, which is 99.5%. The alloy is characterised by high plasticity, but it has low strength properties and, therefore, is not suitable for machining. Moreover, the alloy is corrosion-resistant and has good weldability and high thermal and heat conductivity, which make it suitable for use in the energy and automotive industries [22]. The application of aluminium alloy EN AW-1050A in industry is also influenced by its appearance, which is valued for the glossy surface of the material. In Table 1 the physical properties of the tested aluminium alloy are presented.

The second adherent forming the adhesive joints was non-tempered soda-lime float glass. This type of glass is used in the construction industry and the automotive industry for glazing. Non-tempered glass is a very brittle material, which splits easily and has low impact strength. In order to change the properties of the glass, it is possible to make modifications to increase the strength and flexibility of the glass by applying tempering or bonding of the glass layers with the use of polyvinyl butyral (PVB) foil [24,25]. Modifications are necessary in the automotive industry, where a vehicle is subjected to dynamic forces that can easily damage the glass.

The float glass production process allows for an even sheet of glass with a thickness between 0.4 mm and 25 mm to be obtained [26]. The basic materials included in the composition of the glass selected for the study are as follows: SiO_2_—60–75%, Na_2_O—12–18%, CaO—5–12% [27]. In addition to these materials, soda-lime glass also contains other chemical compounds, which often tint the glass to protect the user from UV radiation, as is the case for car windows. The glass, for the samples selected for testing, was not tinted. The properties of the glass are shown in Table 2.

A two-component epoxy adhesive containing an epoxy resin and curing agent was used to prepare the adhesive joints. The epoxy resin, chosen for the study (trademark: Epidian 5, Ciech Resins, Nowa Sarzyna, Poland) is formed via the reaction of bisphenol A with epichlorodrin [29]. It occurs in the form of a thick liquid with an amber colour. This resin constitutes the basis of adhesives used to bond glass, wood or metal. Table 3 shows the characteristics of the Epidian 5 epoxy resin (Ciech Resins, Nowa Sarzyna, Poland).

The curing agent of the adhesive compound was a polyamide curing agent (trademark: PAC, Ciech Resins, Nowa Sarzyna, Poland). This curing agent is available in a liquid form. It belongs to the group of slow-reacting curing agents and has low reactivity. The adhesive compound made up of the above-mentioned components is characterised by low viscosity, which allows for efficient spreading over the surface of the sample and thorough filling of micro-roughness, ensuring proper mechanical adhesion. In addition, it has high chemical resistance to oils and solvents. Once cured, the adhesive composition exhibits high stiffness. The disadvantage of this composition compared to other available epoxy adhesives is the need for the precise dosage of the resin and curing agent [28,30,31]. The adhesive compound was mixed at a ratio of 80 g of curing agent per 100 g of epoxy resin. A mechanical stirrer with a spindle speed of 460 rpm was used to mix the components to obtain a uniform consistency of the adhesive. The process of curing the resulting adhesive was carried out in stages. The first stage was the gelation of the adhesive compound, which began from the moment of mixing the adhesive components. The gelation time was 180 min at 25 °C. The adhesive compound was then pre-cured. A fully cured adhesive joint was obtained after 7 to 14 days [32].

**Figure 1:**
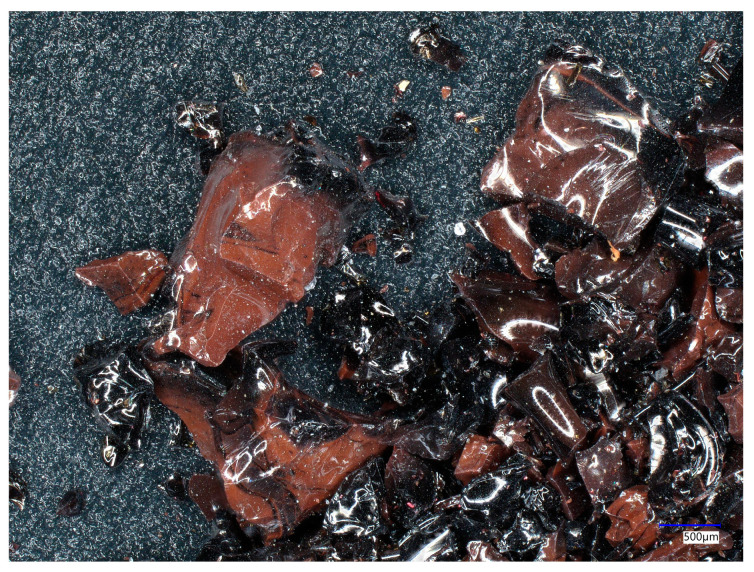
The microscopic view of copper slag.

Two sanding agents, copper slag and glass beads, were used in the study. Copper slag is a by-product of copper ore smelting. The photos were taken at close range using a microscope (model: VHX 500, Keyence, Osaka, Japan). The microscopic view of copper slag is presented in Figure 1.

It is formed by depositing slag on the surface of the molten metal, and then, it is collected for air hardening. The hardening treatment produces granules with sharp, angular edges and a black-red colour [33,34]. Copper slag is widely used as a sandblasting agent due to the easy availability of this material and its low price. Moreover, it is a non-toxic, environmentally friendly material. The hardness of this material is 7 on the Mohs scale. The chemical composition of the material used in the study consisted of the following: SiO_2_ 32.0–46.0%, CaO 18.5–29.0%, FeO 4.5–6.0%, Al2O3 10.0–14.5%, MgO 8.0–12.5% [35]. This material, due to its properties, is widely used as an abrasive; its hardness allows it to be used in copper slag sandblasting to clean mainly metal or concrete surfaces. The grain size of the copper slag used in the study ranged from 0.2 to 0.8 mm.

**Figure 2:**
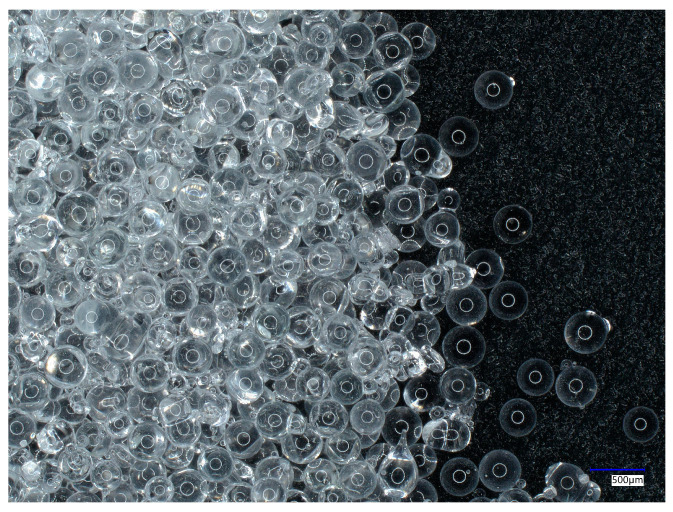
The microscopic view of glass beads.

The second sandblasting agent was glass beads. The microscopic view of glass beads is presented in Figure 2.

This material allows for the very thorough cleaning of metal surfaces and makes them smooth and satin. This agent is suitable to process thin and brittle materials susceptible to surface damage. The glass beads do not cause aggressive surface abrasion of the workpiece, and as in the case of the copper slag, they can be used multiple times. The hardness of glass beads is 6 on the Mohs scale. The chemical composition of the glass beads used in the study was as follows: SiO_2_ 70–73%,Na_2_O + K_2_O 13–15%, CaO 7–11%, MgO 3–5%, Al_2_O_3_ 0.5–2.0% [36]. The size of the glass beads selected for the study ranged from 0.42 to 0.80 mm.

The first stage of the sample preparation was to cut the materials used to a specified size. For the preparation of the EN AW-1050A aluminium alloy samples, a sheet of a 3 mm thickness and dimensions of 2 × 1 m was used. The aluminium alloy sheet was cut into 100 × 25 mm samples using Waterjet Eckert Combo portal cutting machine.

As for the glass samples, samples with dimensions of 125 × 25 mm and a thickness of 4 mm were purchased. The brittleness of the glass, which has not been subjected to any additional treatment that would strengthen the glass structure, does not allow for the strength testing of adhesive lap joints consisting of only 2 elements, in this case a glass sample and an aluminium alloy sample. In overlap joints, the non-tempered glass, under the influence of the forces acting in the strength testing machine, cracks in front of the adhesive layer of the adhesive joint [9]. It is also possible to damage the material because of the stress during the fixing of the adhesive joint in the holder of the strength testing machine.

The glass samples were cut to the length of the overlap. This allowed the glass sample to be placed between the two aluminium alloy samples. The glass samples were cut using a special knife. In order to determine the length of the glass sample, the formula for limiting the overlap length lgr was used. The values of the coefficient of longitudinal elasticity and the thickness of the material were adopted only for glass [37].

(1) $$l_{gr}\;\geq \;5\sqrt{\frac{Egg_{k}}{2G_{k}}}$$

The values are as follows:

*E* = 7.2 ∙ 104 MPa—Young’s modulus of glass

*g* = 4 mm—thickness of glass

*g_k_* = 0.10 mm—adhesive joint layer thickness

*G_k_* = 2200 MPa–shear modulus of the adhesive

The value of the shear modulus of the adhesive in calculating the limiting length of the single-lap was selected for the adhesive formed from the Epidian 5 epoxy resin mixed with the polyamide curing agent in a ratio of 10:6. The calculated limiting length of the overlap was 16.36 mm. The length of the glass sample and, thus, the overlap of the adhesive joint, adopted for the test, was 22 mm. This was due to the difficulty of cutting the sample below 22 mm in length while maintaining the required dimension.

After obtaining the samples of the required dimensions, the focus was made on the surface treatment of adherends. Three groups of samples were selected at this stage.

The first group consisted of samples for which surfaces were untreated. In the second group, the samples were sandblasted with copper slag, and in the third group, they weresandblasted with glass beads.

Sandblasting was carried out manually using a sandblasting gun (Yato Yt-2375, Shanghai, China) and a compressor (Stanley 24l, NU AIR POLSKA Sp. z o.o., Warsaw, Poland). The jet of the sandblasting agent was directed perpendicularly to the surface of the sample. The distance between the nozzle and the treated material was 10 ± 1 cm. The treated sample was inclined at an angle of 45 ± 1° during sandblasting to the work surface. The pressure of the compressor during the sandblasting operation was 0.6 MPa. As for the surface of the aluminium alloy samples, only the overlap part of the specimen was treated. However, the glass samples were sandblasted on both sides. The sandblasting operation of each adherent lasted until a homogeneous structure was obtained on the surface of the samples. During the sandblasting operation, the temperature and humidity were respectively 23 ± 1 °C and 20 ± 1%.

**Figure 3:**
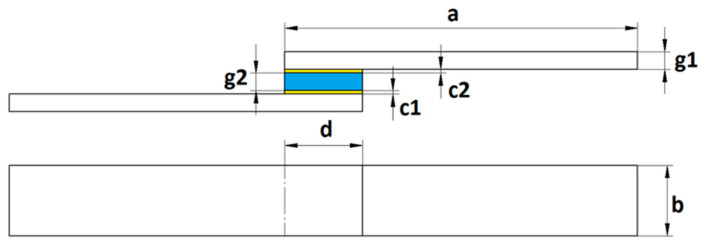
The dimensions of the adhesive joint. a: 100 mm ± 1 mm; b: 25 mm ± 1 mm; g1: 3 mm; g2: 4 mm; d: 22 mm ± 1 mm; c1, c2: 0.1 mm.

Adhesive joints consisting of two of the same aluminium alloy samples and one glass sample were prepared in the study. The design of this joint was determined by the brittleness of the glass, which does not allow a standard overlapping adhesive joint to be created. The purpose of the adopted design was to enable the performance of strength tests without damaging the glass at a location other than the adhesive layer [9]. Figure 3 shows the design of the adhesive joint and the dimensions.

The adhesive joints created in the study differed in the way of surface treatment of the samples. Table 4 shows the configuration diagram of the sample surface treatment with the designation that will be used further on in the article. Configurations A, B and C were divided into joints with the surface of the samples treated by using copper slag and those with the surface of the samples treated by using glass beads. The adhesive joint samples in configuration D were not sandblasted and were therefore the reference variant for both sandblasting agents. Five adhesive joints were prepared for each configuration variant. In total, there were 35 adhesive joints.

The preparation of the adhesive joints was in two stages. The staged way in which the adhesive joints were created resulted from the need to maintain the accuracy of the arrangement of the samples in relation to each other. In the first stage, the focus was on bonding one aluminium alloy sample to the glass. Previously prepared samples were degreased with a Loctite 7063 degreasing agent. The purpose of this treatment was to remove contaminants created during the processing of the materials. The degreasing process was carried out by applying the degreasing agent to the surface of the sample and then wiping it in a single motion using a dust-free swab. This action was repeated twice. In the third degreasing of the sample surface, the cleaning agent was left to evaporate.

The prepared adhesive was applied to the surface of the aluminium alloy, and then, the aluminium alloy sample was combined with the glass sample. The resulting joint was left to pre-cure for a period of 48 h. During the curing process, the joint was pressed with a 0.04 MPa.

In the second stage of the adhesive joint preparation, the second sample of the aluminium alloy was bonded to the glass. This stage started after the pre-curing time had elapsed. The process of cleaning the samples and joining the components followed the same scheme as described before. The adhesive joints consisting of two aluminium samples and a glass sample were left for a period of seven days to allow for a full cure of the adhesive. The pressure of 0.04 MPa was also used at this stage. During the preparation of the adhesive joints and their cure, the temperature and humidity were, respectively, 23 ± 1 °C and 20 ± 1%.

### 2.2. Strength Test

The prepared adhesive joints were subjected to shear strength tests. For this purpose, the Zwick/Roell Z150 testing machine was used. The tests were carried out in accordance with the norm EN 1465 [38]. The speed of the test was 20 mm/min. The following conditions prevailed during the strength tests: temperature, 22 ± 1 °C; humidity, 23 ± 1%. The adhesive joints subjected to the strength test did not reveal any damage to the glass during the test.

After the shear strength test, the failure mode of the adhesive joints was analysed. In determining the type of the failure mode, the norm ISO 10365 was used [39].

### 2.3. Surface Roughness Test

One sample, from each type of the prepared glass and aluminium alloy samples, was selected for the analysis of the structure of the surface topography. This study allowed the changes occurring on the surface of the samples to be described before and after the sandblasting treatment. In order to carry out the analysis, the 3D profilographometer (Hommel-Etamic T8000 RC120–400, Villingen-Schwenningen, Germany) was used. The following 3D roughness parameters were used for the analysis [40]:

- Sq—mean square deviation of the height of the surface unevenness from the reference plane;
- Sp—maximum height of surface elevations;
- Sv—maximum depth of the valley surface;
- Sz—maximum height of the 3D surface profile;
- Sa—the arithmetic mean deviation of the height of surface unevenness from the refer-ence plane.

### 2.4. Methods of Statistical Analysis

The aim of the statistical analysis was to compare the shear strength of the adhesive joints with regard to the prepared surface of the samples and to demonstrate the effect of changing the adhesive surface of the samples on the strength of the joints. In order to perform the statistical analysis, a multi-dimensional analysis of variance test (MANOVA) was used, which allows for an analysis of variance of multiple dependent variables against any number of co-variables. MANOVA requires the following conditions [41]:

1. The study groups show a normal distribution;
2. The variance between groups is the same;
3. The results of the trials are taken randomly;
4. The studied variables are measurable.

The Shapiro–Wilk test and the Bartlett test were used to satisfy the conditions for multidimensional variance. The first statistical test is one of a number of tests allowing the normal distribution of the studied sample to be verified. Its advantage in this study is that it is recommended for testing samples with a small number of elements. It is also characterised by a high test power [42].

The second statistical test checks the homogeneity of the variance between the studied groups. It allows the homogeneity of variance to be tested on more than 2 groups. This test compares the weighted arithmetic mean of the variance with the weighted geometric mean of the variance. Where the variances compared are not different from each other, the arithmetic mean is equal to or less than the weighted mean [41,42]. The assumed significance level for all statistical tests performed is *α* = 0.05.

## 3. Results

### 3.1. Surface Topography and Surface Roughness Parameters of Adherends

The surface roughness results, together with topography maps of the surface of adherends and surface roughness parameters, are presented in Table 5 and Table 6. Each table is divided in terms of the surface preparation of the selected material.

Table 5 shows the surface topography before and after the sandblasting of EN AW-1050A aluminium alloy samples. The lowest value of the Sa surface roughness parameter is shown based on the untreated surface. This surface is covered by a unidirectional pattern of micro-roughness. The topography map shows small elevations, which are a defect in the material itself.

The sandblasted surfaces do not show a directed structure. The largest changes in surface roughness were obtained for the sample sandblasted with copper slag. In the case of this sample, the increase in the Sa surface roughness parameter relative to the non-sandblasted surface is 2130%. The difference in the Sz surface roughness parameter between these surfaces is 39.62 µm. On the basis of the observation of the surface topography maps, it can be concluded that elevations on this surface are not homogenous. The areas with lower heights of microroughness are visible.

The value of the Sz surface roughness parameter for the surface prepared with glass beads is half the Sz surface roughness parameter value for the surface prepared with copper slag. The increase in the value of the Sa surface roughness parameter in relation to the unmachined surface is 1403%. The height of the elevations on the surface prepared by using glass beads is uniform.

The surface of the glass sample, which was not treated mechanically, has no characteristic features. Only one defect is visible, which contributes to the rise of the value of the Sz surface roughness parameter. The value of the Sa surface roughness parameter for this topography is close to zero. The difference in the values of Sp, Sv and Sz surface roughness parameters between the pre- and post-treatment surfaces is significant. This is due to the brittle structure of the glass. The highest value of the Sa surface roughness parameter was obtained for the surface prepared by sandblasting with glass beads. The geometric structure of this surface is varied. There are islands with clusters of hills or valleys. Despite the higher value of the Sa surface roughness parameter in relation to the same parameter for the copper slag, the value of the Sz surface roughness parameter is lower by 7 µm.

In the case of the surface treatment with the copper slag, a homogeneous surface topography can be observed. The figure in Table 6 reveals that the heights of hills and valleys are similar. Also, their arrangement on the surface is regular. The value of the Sp surface roughness parameter for sandblasting with the copper slag in comparison to the same process with the glass beads is lower by 11.20 µm, while the Sv surface roughness parameter is higher by 18.20 µm. However, when analysing the view of the surface topography, a characteristic recess different from others can be noticed. This recess contributes to an increase in the value of the Sv surface roughness parameter.

### 3.2. Strength Results

**Figure 4:**
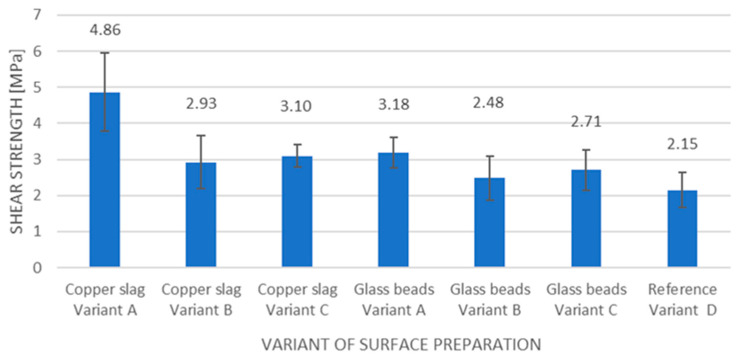
Graph of the strength of adhesive joints.

The results obtained for the strength of the adhesive joints are shown in the graph in Figure 4.

The results of the strength of the adhesive joints prepared in variants A-C using copper slag and glass beads and the reference joints prepared in variant D are shown in Figure 4. In the diagram, to distinguish the surface preparation of the samples, each type of adhesive joint is marked with the sandblasting agent and the type of preparation variant.

The first group of adhesive joints included adhesive joints for which the surface of the sample was prepared using copper slag. In this group of adhesive joints, an increase in strength, compared to adhesive joints prepared in variant D, can be observed. In this group, the adhesive joint prepared in variant A has the highest strength. The percentage increase in the strength of this variant in relation to the adhesive joint prepared in variant D is 126%. The lowest strength value in this group of adhesive joints is obtained for the adhesive joint prepared in variant B. The percentage difference between adhesive joints prepared in variants A and B is 66%. Comparing adhesive joints prepared in variants B and C, it is apparent that the strength value for the adhesive joint prepared in variant C is higher than for the adhesive joint prepared in variant B.

The second group of adhesive joints included adhesive joints for which the surface was prepared using glass beads. In this group, an increase in strength, relative to the adhesive joints prepared in variant D, was also observed. The adhesive joint with variant A had the highest strength. The percentage increase in the strength of the adhesive joint treated in variant A in relation to the adhesive joint treated in variant D is 48%. The lowest value of the shear strength in this group was obtained using the adhesive joint prepared in variant B.

Comparing the strength results of all adhesive joints presented in Figure 4, the highest strength was gained by the adhesive joints in variant A prepared using copper slag.

### 3.3. Results of Normal Distribution and Homogeneity of Variance

The statistical analysis was carried out on the adhesive joints prepared in variants A, B and C. The statistical calculations of the normal distribution performed with the Shapiro–Wilk test are presented in Table 7. It shows the test probability *p*-value with parameters *n* = 4 and *α* = 0.05.

The analysis of the results of the Shapiro–Wilk test allows us to conclude that there are no grounds to reject the zero hypothesis on the basis of the obtained values of test probability for all of the studied samples. There are no statistically significant differences.

Bartlett’s test of homogeneity of variance is presented in Table 8. The table includes the values calculated using the Bartlett test, the value of the degrees of freedom and the test probability level.

On the basis of the homogeneity of variance test performed, it can be concluded that there is no basis for rejecting the zero hypothesis, which concerns the existence of the homogeneity of variance. The test probability value *p* is greater than the level of statistical significance.

The completed statistical tests for normal distribution and homogeneity of variance allow us to carry out the test of multivariate analysis of variance.

### 3.4. Analysis of Variance

The values of the results of the MANOVA carried out are shown in Table 9. The statistical analysis performed compares the strength values of the adhesive joints for the three factors:

- Between the sandblasting agent used;
- Between the type of adhesive surface treatment;
- Between the sandblasting agent used and the type of adhesive surface treatment.

Significant statistical differences were observed for two parameters, the type of sandblasting agent and the joint preparation variant. A lack of statistical significance was observed in the case of the parameter comparing the type of sandblasting agent used and the sandblasting variant. This indicates that there is a statistical similarity. However, the *p*-value of 0.069 for this parameter is on the verge of accepting the test as statistically insignificant. In order to verify the strength similarities between the adhesive joints in detail, Tukey’s POST-HOC test was prepared (Table 10).

The POST-HOC test (Table 10) showed that statistical significance between the other adhesive joints occurred only for adhesive joints with the surfaces of samples treated by copper slag in variant A. The other types of adhesive joints did not achieve statistical significance, from which it can be concluded that the strength of that adhesive joints is similar.

## 4. Discussion

This paper focuses on comparing the strength of adhesive joints formed from different materials, which were glass and the EN AW-1010A aluminium alloy. Choosing the right method of surface preparation of both materials to be joined so that the adhesive joint is characterised by the highest possible strength requires a thorough analysis of the surface topography and physicochemical properties. The research carried out showed that the application of abrasive blasting to the materials tested improves the properties. On the basis of the results obtained, it is not possible to clearly state which surface roughness parameters influence the increase in the strength of the adhesive bond. It is only noticeable that the adhesive joint preparation in variant A, where the surface of both materials was prepared by sandblasting with copper slag, obtained the highest strength and is the only one statistically different from the other adhesive joint preparation variants. This shows that, when using epoxy adhesive, abrasive treatment should be applied to both bonded surfaces in glass–aluminium alloy adhesive joints. It is noticeable that variant A in adhesive joints, for which the surface was prepared using glass beads, did not achieve similar values to variant A prepared using copper slag. Despite the higher values of the roughness parameters (Sq, Sp, Sv, Sz, Sa), statistically, the strength of the adhesive joints of variant A prepared with glass beads is similar to the other adhesive joints. This may be indicative of the fact that the spherical structure of the glass beads creates micro-roughness on the surface, which prevents proper attachment of the adhesive bond and thus weakens the mechanical adhesion. In the case of the post-copper route, the shape of the abrasive grains is varied and has many sharp edges.

The failure analysis of the adhesive joints showed no cracks in the glass. There was only adhesive failure, where the adhesive was in contact with the glass sample. In addition, none of the glass sample surfaces were shown to have greater mechanical adhesion. The detachment of the adhesive layer from the selected glass surface was random.

Despite the increase in the strength of the adhesive joints after sandblasting on the surface of the glass sample, it is worth noting that the use of the selected abrasives negatively affects the glass structure, significantly weakening it. Further research could include changing the abrasive to a softer abrasive.

## 5. Conclusions

No cracks appeared on the glass samples during the adhesive bond break tests. This means that the proposed design of the adhesive joints allows the appropriate shear strength results of the adhesive joints with the use of glass to be obtained.

The use of sandblasting for the surface treatment of glass and aluminium alloy adherends results in an increase in the shear strength. The surfaces sandblasted with both copper slag and glass beads differed from untreated surfaces. The aluminium alloy samples achieved the highest value of the Sq surface roughness parameter after sandblasting with copper slag. The percentage difference of the Sq surface roughness parameter between the sandblasting agents for the aluminium alloy was 56%. In the case of the glass samples, the highest value of the Sq surface roughness parameter was obtained after sandblasting the samples with glass beads. The percentage difference of the Sq surface roughness parameter between the sandblasting agents for this material was 64%.

On the basis of the strength tests performed, it was found that irrespective of the sandblasting agent, the highest value of the adhesive strength is characteristic of the adhesive joints where both adherends were prepared using sandblasting. Of all the adhesive joints examined, the highest strength was obtained using the adhesive joints for which the adherent surface was prepared by sandblasting with the copper slag (Variant A).

Manova showed a lack of statistical significance for the results of the adhesive joint strengths compared in terms of the sandblasting agent and the adhesive joint variant. However, on the basis of Tukey’s test, it is evident that the strength of the adhesive for which the adherent surface was prepared by sandblasting with the copper slag (variant A) is statistically significant as compared to the other adhesive joints.

## Figures and Tables

**Table 1:** Physical properties of aluminium alloy EN AW-1050A [23].

Property	Value
Density	2.70 g/cm^3^
Modulus of elasticity E	69,000 MPa
Shear modulus G	25,900 MPa
Poisson’s number	0.33
Thermal conductivity	229 W/mK
Melt temperature	658 °C
Solidification temperature	645 °C

**Table 2:** Physical properties of non-tempered soda-lime glass [28].

Property	Value
Density	2.500 g/cm^3^
Modulus of elasticity E	72,000 MPa
Poisson’s number	0.22
Maximum working temperature	110 °C
Solidification temperature	645 °C

**Table 3:** Characteristics of the epoxy resin Epidian 5 [30].

Feature	Value
Density at 25 °C	approx. 1.7 g/cm^3^
Viscosity at 25 °C	20,000–30,000 mPa s
Epoxy number	0.48–0.51 mol/100 g

**Table 4:** Adhesive joint configuration diagram.

Sandblasting Variant	Cooper Slag		Glass Beads	
	Type of Material Treated			
	Glass Beads	Aluminium Alloy	Glass Beads	Aluminium Alloy
A	+	+	+	+
B	+	−	+	−
C	−	+	−	+
D	−	−	−	−

(−)—treatment not used; (+)—treatment used.

**Table 5:** Surface roughness of aluminium alloy samples.

**Without Sandblasting**		
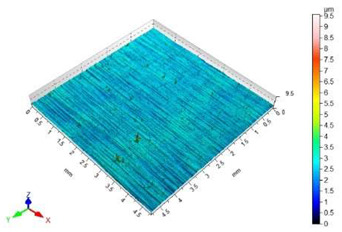	Sq	0.199 µm
	Sp	6.98 µm
	Sv	2.60 µm
	Sz	9.58 µm
	Sa	0.139 µm
**Sandblasting with Copper Slag**		
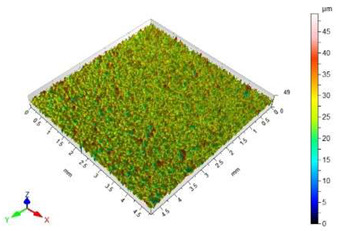	Sq	4.10 µm
	Sp	23.10 µm
	Sv	26.10 µm
	Sz	49.20 µm
	Sa	3.10 µm
**Sandblasting with Glass Beads**		
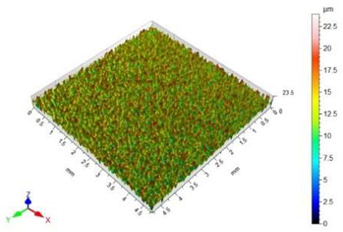	Sq	2.62 µm
	Sp	11.20 µm
	Sv	12.70 µm
	Sz	23.90 µm
	Sa	2.09 µm

**Table 6:** Surface roughness of glass samples.

**Without Sandblasting**		
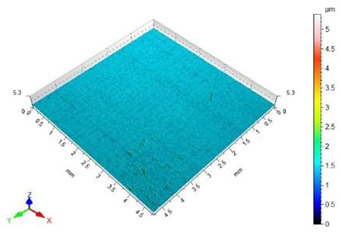	Sq	0.035 µm
	Sp	3.91 µm
	Sv	1.48 µm
	Sz	5.39 µm
	Sa	0.019 µm
**Sandblasting with Copper Slag**		
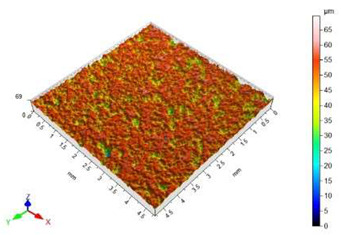	Sq	5.14 µm
	Sp	19.10 µm
	Sv	50.50 µm
	Sz	69.60 µm
	Sa	3.90 µm
**Sandblasting with Glass Beads**		
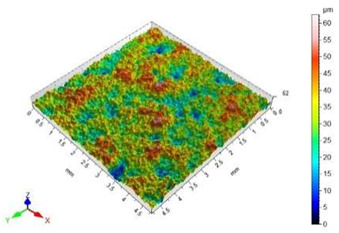	Sq	8.43 µm
	Sp	30.30 µm
	Sv	32.30 µm
	Sz	62.60 µm
	Sa	6.69 µm

**Table 7:** Shapiro–Wilk test results. Assumption: H_0_: the tested sample has a normal distribution; H_1_: the tested sample does not have a normal distribution; *α* = 0.05.

Sandblasting Agent	Variant	*p*-Value
copper slag	A	0.447
	B	0.113
	C	0.161
glass beads	A	0.942
	B	0.938
	C	0.505

**Table 8:** Bartlett test results. H_0_: the tested sample has a homogeneity of variance; H_1_: the tested sample does not have a homogeneity of variance; *α* = 0.05.

Bartlett	Df	*p*-Value
6.658	5	0.247

**Table 9:** MANOVA test results.

Tested Group	Df	Sum of Squares	*F*-Value	*p*-Value
Use of the sandblasting agent	1	5.284	11.824	0.002
Type of surface treatment	2	10.080	11.279	0.000
Use of the sandblasting agent: type of surface treatment	2	2.679	2.997	0.069

**Table 10:** POST-HOC test results.

Comparison Pairs	*p*-Value
glass beads A—copper slag A	0.006
copper slag B—copper slag A	0.002
glass beads B—copper slag A	0.000
copper slag C—copper slag A	0.004
glass beads C—copper slag A	0.000
copper slag B—glass beads A	0.989
glass beads B—glass beads A	0.578
copper slag B—glass beads A	0.999
glass beads C—glass beads A	0.869
glass beads B—copper slag B	0.898
copper slag C—copper slag B	0.998
glass beads C—copper slag B	0.995
copper slag C—glass beads B	0.693
glass beads C—glass beads B	0.994
glass beads C—copper slag C	0.935

## Data Availability

The original contributions presented in this study are included in the article. Further inquiries can be directed to the corresponding author.
